# Taxing experiences: theorizing negative incidents at the frontlines

**DOI:** 10.1080/14719037.2024.2384463

**Published:** 2024-08-02

**Authors:** Shelena Keulemans, Dorian Schaap

**Affiliations:** Department of Public Administration, Institute for Management Research, Radboud University, Nijmegen, The Netherlands

**Keywords:** Frontline workers, negative incidents, framework development, abduction, tax administration

## Abstract

Negative interactions with citizens can leave frontline workers stressed, anxious, and traumatized. Here, we construct a theoretically interdisciplinary framework for understanding frontline negative incidents that was thus far lacking. Drawing from 1302 descriptions of Dutch and Belgian frontline tax officials’ most negative experience with citizens, we identify four overarching categories: citizen aggression, facing tragedy, violations of professional norms, and absence of support. We show the importance of non-aggression incidents and reveal how the nature, direction, and impact of these incidents differ and depend on workers’ sense-making processes. We outline consequences for the study of frontline encounters and for public agencies.

## Introduction

‘The guy takes out a knife [and] started cutting himself […] He kept swallowing pills. This stuff went on an hour and a half. But I didn’t want him to kill himself there’ (welfare agent in Dubois 2010, 166–167). Particularly negative interactions with citizens impact frontline workers on a fundamental level (cf. Davidovitz and Cohen 2021; Sivis-Cetinkaya 2015; Tzafrir, Enosh, and Gur 2015). They can cause stress, anxiety, or trauma (e.g. Dubois 2010). To protect themselves, frontline workers can adopt adaptation strategies that may trigger a vicious cycle of negative incidents, such as adopting alternative work modes (Zacka 2017) that increase their chances of being met with citizen aggression (Friis et al. 2020; Muir 1977).

Yet current scholarship on frontline negative incidents remains disparate and scattered. Rich insights into frontline incidents can be drawn from in-depth, qualitative inquiries that take a narrative, sense-making approach to citizen-state interactions (e.g. Dubois 2010; Maynard-Moody and Musheno 2003; Raaphorst 2017, 2018; Zacka 2017), but their emphasis on the unique nature of each interaction comes at the cost of understanding common patterns. Larger-scale studies, by contrast, tend to examine frontline interactions by merely studying *hypothetical* citizen behaviour and/or frontline workers’ hypothetical coping response to that behaviour (e.g. Braithwaite 2009; Nielsen and Nielsen 2022; Nielsen, Nielsen, and Bisgaard 2021). Hypothetical foci are insufficient to understand actual interactions between frontline workers and citizens and how these affect frontline workers. Moreover, coping captures *responses to* rather than *what happens in* frontline interactions.

Consequently, we lack insight into the citizen interactions that frontline workers *themselves* identify as negative incidents and how these impact them. How can we understand frontline negative incidents? And how are they experienced in a public agency? Answering these questions helps us understand what characteristics and dynamics turn a frontline interaction into a negative incident, which is essential for their study and prevention. Taking an abductive approach, we address this gap by proposing a negative incident framework (cf. Richardson and Kramer 2006). We integrate theoretical insights from organizational psychology, public administration, criminology, and sociology with empirical findings from 1302 negative incidents experienced by a cross-national sample of frontline tax officials.

Theoretically, we offer a comprehensive, theoretically grounded interdisciplinary framework for understanding frontline negative incidents that enables future theorizing and operationalization; insights into how negative incidents manifest themselves empirically in public agencies; and knowledge of which negative incidents affect frontline workers the most. Combined, these contributions provide invaluable building blocks for developing a theory of frontline negative incidents.

Practically, our framework provides public agencies with much-needed guidance on how they can help frontline workers deal with negative incidents or avoid their occurrence altogether, improving workers’ mental health and aiding them in maintaining a ‘pragmatic humanism’ (Björk 2008) in their dealings with citizens, even in the face of regular disillusion (Blau 1960).

After outlining our research setting, data collection procedure, and abductive approach to framework development, we theoretically substantiate our incident categories and analyze empirically how those categories manifest themselves in public agencies. Finally, we discuss the framework’s implications and future research avenues.

## Research setting: Dutch and Belgian frontline tax officials

Frontline workers, an analytically distinct category of public servants, share structurally similar work conditions, supporting the theoretical generalizability of research into their various types (Keulemans 2020). These similarities include their face-to-face engagement with citizens to deliver public services, their considerable discretion, and their constraints due to limited resources. Their decisions can strongly impact citizens’ lives (Lipsky 2010).

This study was conducted in the Dutch and Belgian tax administration, among frontline tax officials who audit small or medium-sized enterprises (SMEs). Performing similar tasks in both countries (Keulemans 2020), these officials conduct audits on-site, visiting either the enterprise or an entrepreneur’s home. Their responsibilities are multifarious and sometimes conflicting as they balance service-oriented tasks, such as citizen education or behaviour modification, with law enforcement (Van de Walle and Raaphorst 2019). Diversity in client characteristics and task attributes (cf. Keulemans 2020) foster the potential generalizability of our framework.

After being assigned a case, the tax official contacts the entrepreneur, often by phone, to announce the audit and plan a preliminary meeting. Entrepreneurs receive a confirmation letter stating the meeting date and the documents the entrepreneur needs to present. During that on-site meeting, officials explain why the entrepreneur is audited and how the audit will proceed. The official and entrepreneur discuss the enterprise and its operational practices to facilitate the audit, often with the entrepreneur’s accountant or tax advisor present.

The tax official then audits bookkeeping records, checking for inconsistencies, mistakes, missing data, or other issues. Tax officials generally present their conclusions in a final meeting with the entrepreneur (and, optionally, the tax advisor) during which entrepreneurs can clarify or explain audit findings. If these explanations seem implausible or fail to fully account for the issues, tax officials will take corrective action, considering whether mistakes stemmed from ignorance, negligence, or intent.

Tax officials’ discretion and the high stakes involved can motivate citizens to try to influence frontline interactions and outcomes. Dynamics of defiance, resistance, and posturing are particularly salient (Braithwaite 2009), increasing the likelihood of negative incidents occurring in tax officials’ frontline work. This makes tax agencies useful cases for framework development.

## Collecting incident data

The potentially sensitive nature of negative incidents necessitated an anonymous, digital survey to minimize social desirability (cf. Krosnick, Judd, and Wittenbrink 2005; Schwarz 2008). All Dutch and Belgian tax officials in the SME domain were invited to participate in the summer of 2016,^1^ yielding a primary sample of 4639 frontline tax officials. The response rate was 42.2% (*N* = 1959). We subsequently included tax officials who confirmed having face-to-face citizen contact (*N* = 1584) and who had given any reply to the negative incident measures (*N* = 1405). Lastly, we omitted respondents with unanalysable answers, such as random letters or punctuation marks, explicit refusals to answer the question (nine cases), some respondents saying they did not know whether they had such experiences or the question was not applicable, and a few mistakenly mentioning positive incidents. In the final sample (*N* = 1302), the mean age was 51.5 years. 68.8% were male (*N* = 896), and 30.2% were female (*N* = 393). 58.8% were Dutch (*N* = 766), 41.2% were Belgian (*N* = 536). The average tenure was 25.2 years.

To collect negative incidents, we drew from the critical incident technique [CIT] (Flanagan 1954). CIT is a retrospective, qualitative research approach that invites respondents to self-report vividly recalled meaningful occurrences (Papouli 2016). It is often applied to recollections of particularly negative or positive interaction incidents (Edvardsson and Roos 2001, 253). Written incident accounts such as these have been demonstrated to offer high-quality, descriptive data that are more focused and reflective than oral accounts (Handy and Ross 2005, 40).

This yielded an open-ended survey question that asked respondents: ‘Think back to the most negative experience you have had with a taxpayer during your entire career with the tax administration and describe that experience in the survey text box. What happened? What did the taxpayer do to make this the most negative experience of your career with the tax administration?’ The latter two probing questions were to encourage respondents to provide a detailed description of the incident.

To assess incident impact, respondents were then asked to indicate how much of an impact the described incident had on them by dragging a slider between ‘no impact at all’ (0) and ‘a very big impact’ (100).

## An abductive approach to developing a negative incident framework

Abductive approaches attempt to develop useful explanations for an observed phenomenon. It assumes that ideas or theories cannot be found within the data, but are heuristic tools based on pre-existing knowledge or practical experience that can usefully be applied as a preliminary analytical framework (Bowen 2006; Richardson and Kramer 2006). In abduction, empirical data is explained ‘through the elaboration, modification, or combination of pre-existing concepts’ (Kelle 1995, 34). Inherent to an abductive approach is a continual interplay between data, data interpretation, and theory (Bowen 2006).

Respondent answers were disparate, including a wide range of situations, encounters and events, necessitating the process of abduction. For instance, while much scholarly and media attention goes to violence against public workers (e.g. Bishop, Korczynski, and Cohen 2005; Schat and Kelloway 2005), only a minority of described incidents referred to physical assault. A larger number of responses even concerned negative incidents that were not directly related to the actual citizen encounter that the question referred to. Clearly, a framework was needed that made sense of these contra-intuitive findings. So, through an analytical process of constant comparison (Thompson Burdine, Thorne, and Sandhu 2021; Grove 1988), preliminary interpretations of the findings were tested and challenged (Thorne, Reimer-Kirkham, and O’Flynn-Magee 2004) and category definitions and rules emerged in several cycles of dialogue between the two authors (Grove 1988).

First, we developed several rough, a priori categories of negative incidents based on our pre-existing domain knowledge of various streams of literature (Richardson and Kramer 2006). With these conceptions, the authors scanned a random subsection of the data, 300 negative incident responses [NIs], ‘[A]ssociating data with ideas’ (Richardson and Kramer 2006, 500). This led to an emergent negative incident framework (v0) with seven preliminary categories: personal attacks; complaints; manipulation and lies; avoidance behaviour; personal tragedy; colleagues and supervisors; other/none.

Proceeding this way, the provisional framework was then adjusted based on its application to another subsection of 300 NIs, leading to a full framework (v1). v1 featured a more nuanced distinction between different forms of personal attacks (i.e. threats and assaults; insinuations and intimidation; outbursts and insults). A research assistant then provisionally coded all incidents based on v1. Both authors subsequently coded a small subsection of the data (100 NIs) and compared the results. Not unexpectedly given the open-ended nature of our data and the disparate responses that were given (Montgomery and Crittenden 1977), our assessments showed some divergence.

Rather than attempting to align our judgements, which would ‘force fit’ data to theory (Jackson and Trochim 2002), we reconfigured our framework to better fit the complexity of the multifaceted theoretical framework and data. Reconfigurations included reclassifying preliminary incident categories as belonging to a common denominator, splitting up some categories because they were critical incidents for different reasons, and expanding some categories to more comprehensively capture the types of occurrences that tax officials experienced as critical incidents. This led to a reframed framework (v2).

Some elements of v2 proved overly faithful to their theoretical origins: further adjustment was required to do justice to the constellation of involved actors and their motivations. This was apparent in translating workplace psychological aggression literature – which usually refers to colleagues or managers as culprits – to the context of citizen-frontline worker interactions (with their different social dynamics and disparate physical spaces). Similarly, the purpose or direction of various forms of unpleasant citizen behaviour proved a point of contention: were they expressive or instrumental in nature? These observations merited some final, text-based, finetuning (Popping 2015). This resulted in a third and final framework (v3, see Appendix I) with four overarching negative incident categories:

- citizen aggression;
- facing tragedy;
- violations of professional norms;
- absence of support.

To substantiate our negative incident framework, we first conceptualize each incident category. We then deepen that theoretical understanding by empirically demonstrating how each category manifests itself in frontline workers’ lived experiences. To that end, we coded and analyzed all negative incident recollections using v3 as a coding scheme.

## Theorizing the negative incident categories

To theorize the negative incident categories, we combine insights from several academic disciplines. Organizational psychology is especially focused on notions of workplace aggression (e.g. Schat and Kelloway 2003) and social safety and support in the workplace (e.g. Bishop, Korczynski, and Cohen 2005). Public administration is concerned with the complexities and tensions inherent to street-level bureaucracy, including undesirable citizen behaviour (e.g. Dubois 2010; Nielsen and Nielsen 2022). In criminology, danger and violence against public officials is a key field of interest (Paoline 2003; Van Reemst and Fischer 2019). And (sociological) literature on specific frontline professions such as social work, policing and nursing often takes an interdisciplinary angle towards its subject profession (e.g. Coetzee and Klopper 2010; Savaya, Gardner, and Stange 2011), at the expense of different professions’ shared characteristics. Drawing from these fields allows us to contrast various types of incidents *and* shed light on the dynamics of situations and people that turn interactions into negative incidents. Below, we show how the four negative incident categories are understood in previous research, before fleshing them out empirically through tax officials’ lived experiences.

### Citizen aggression

Citizen aggression is the most recurrent framework category in the literature due to its prevalence and negative consequences for frontline workers and their organization (e.g. Dubois 2010; Keesman and Weenink 2020). Analogous to Schat and Kelloway’s (2005, 191) definition of workplace aggression, we conceptualize citizen aggression as ‘citizen behaviour that occurs within or outside the public agency that is intended to physically or psychologically harm a frontline worker […] and occurs in a work-related context’. Aggression incidents can occur outside the primary workplace, for instance during on-site visits (Schat and Kelloway 2005). The behaviour, moreover, is *meant* to hurt; while it can be impulsive, it is goal-oriented.

We distinguish between physical and psychological aggression. *Physical aggression* refers to instances of physical assault and threats of assault. Actual assault and threats of assault trigger similar physical and emotional responses that include stress reactions, fear, depression or anxiety (e.g. Keesman and Weenink 2020; Rogers and Kelloway 1997). As a result, threats alone can be enough to incite behavioural change in frontline workers (e.g. Muir 1977; Skolnick 1966).

By contrast, psychological aggression is ‘a verbal or symbolic act, the typical immediate effect of which is psychological harm’ (Schat and Frone 2011, 24). These acts – including yelling, swearing, making spiteful comments, and intimidation – trigger fear, anxiety, and alienation that are likely to deplete frontline workers’ resources (cf. Schat and Frone 2011). Psychological aggression may cause frontline workers to either refrain from discretionary behaviour in the future or use their discretion to satisfy the violator’s demands (Davidovitz and Cohen 2021; Dubois 2010). Both adaptation strategies can obstruct effective and just frontline work.

### Facing tragedy and the inability to make a difference

Literature across disciplines acknowledges that frontline workers encounter human tragedy (e.g. Dubois 2010; Muir 1977; Van der Ploeg, Dorresteijn, and Kleber 2003). They often deal with vulnerable citizen groups and with situations where clients cannot hide their suffering, despair or loss of dignity (e.g. Dubois 2010). For some frontline workers, their very responsibility is to deal with tragedy: paramedics, police officers or judges (e.g. Regehr, Goldberg, and Hughes 2002; Van der Ploeg, Dorresteijn, and Kleber 2003). For others, like social workers, nurses or teachers, a phenomenon so common that it might as well be their responsibility too (e.g. Dubois 2010; Maynard-Moody and Musheno 2003; Mousa et al. 2023). Even frontline workers who do not professionally need to address human tragedy, will through the sheer number of people they encounter face unfiltered suffering occasionally (e.g. Sivis-Cetinkaya 2015).

Literature on frontline professions often frames citizen tragedy as an ethical dilemma or form of action uncertainty for frontline workers rather than identifying tragedy as a negative incident for frontline workers *themselves* (e.g. Savaya, Gardner, and Stange 2011; Sivis-Cetinkaya 2015; cf.; Raaphorst 2018). Yet facing citizen tragedy can profoundly affect frontline workers. They can rarely solve tragedy; often, they can merely ameliorate its consequences, buy time, or show empathy (Maynard-Moody and Musheno 2003; Muir 1977). This discrepancy between the frontline workers’ limited resources and the scale of human suffering they sometimes encounter, implies a strain between what they can realistically do and what they feel the situation requires (cf. Tzafrir, Enosh, and Gur 2015; Zacka 2017), resulting in feelings of distress, guilt or trauma (Regehr, Goldberg, and Hughes 2002). Caring about citizens hence has a cost: it can lead to compassion fatigue (Figley 1995) – compassionate energy that is expended surpasses restorative processes, causing exhaustion and withdrawal (Coetzee and Klopper 2010). This form of psychological distress is relatively common among frontline workers (Sciepura and Linos 2022).

### Violations of professional norms

This category captures a variety of undesirable citizen behaviours that violate frontline workers’ professional norms: frontline work is guided by psychological contracts (e.g. Davidovitz and Cohen 2021) based on shared expectations of proper conduct in the professional setting. If citizens breach this contract, it constitutes a violation of professional norms (Davidovitz and Cohen 2021) that may turn the bureaucratic encounter into a negative incident (Savaya, Gardner, and Stange 2011).

We distinguish between instrumental and expressive violations of professional norms. *Instrumental violations* are committed because a citizen wants to gain something. To do their job, frontline workers require citizens’ honesty and compliance (Keulemans and Van de Walle 2020b). Dishonest citizens violate frontline workers’ ethical and moral principles (Davidovitz and Cohen 2021), making workers feel naïve and foolish (Blau 1960). Similar discomfort arises when frontline workers feel pressured to violate their *own* ethical principles (Dubois 2010; Savaya, Gardner, and Stange 2011). This happens when citizens illegitimately try to positively affect the outcome of their encounter, for instance by offering bribes or pressuring workers to break rules on their behalf (Keulemans and Van de Walle 2020b).

Professional norms are also violated when citizens voice their displeasure with the frontline worker in an inappropriate or unacceptable way. This can be direct when the worker is (illegitimately) accused of unjust treatment, wrong decisions, or ill intent. Yet more underhandedly or indirectly, the citizen could adhere to all rules of professional engagement during the interaction, only to file a complaint later or badmouth the worker to their manager (Davidovitz and Cohen 2021). Such painful and sometimes traumatizing attempts to damage the frontline worker’s reputation can be meant to publicly shame them, suggesting an instrumental purpose (De Boer 2021).

*Expressive violations* refer to citizens breaking more implicit expectations that frontline workers have, such as citizens having a cooperative attitude or using polite language. Citizens can first violate these norms through unexpected displays of emotion. Sociological and street-level bureaucracy studies show that citizen conduct is not as bound by bureaucratic rules as that of the frontline worker. This can make their actions, even when they are not intended to hurt the worker, disconcertingly unpredictable (Dubois 2010; Skolnick 1966). Citizens unexpectedly showing emotion, such as indignation or sadness, can cause severe discomfort (Raaphorst 2018). Especially (short-lived) emotional outbursts could become negative incidents (Bishop, Korczynski, and Cohen 2005; Dubois 2010).

Second, implying a more systematic expressive norm violation, comes the notion of ‘the asshole’ (Van Maanen 1978), a label from the intersection of sociology and criminology ‘of sufficient importance to merit its own category’ (Crank 2014, 95) of citizen behaviour vis-à-vis frontline workers. Citizens who behave like assholes are not dangerous or violent and may not have broken any law or agreement (Bittner 1967; Sausdal 2018; Van Maanen 1978, 315). However, by challenging the frontline worker’s authority and by being argumentative and uncooperative (Crank 2014; Van Maanen 1978), they are considered ‘ungovernable’ (Muir 1977). Assholes refuse to share the worker’s definition of a situation (Van Maanen 1978, 309). This lack of common ground makes them difficult and unpleasant to deal with (Sausdal 2018; Van Maanen 1978). Since their motives are not understood, assholes are simply assumed to be driven by the desire to give the worker a hard time (Bittner 1967, 709).

### Absence of support at work

Our framework theorizes negative incidents that occur *during* citizen-frontline worker interactions. Yet our abductive process revealed that the absence of collegial or supervisory support following an unpleasant citizen interaction can be what turns that interaction into a negative incident, warranting its inclusion as a standalone category. Consequences of absent support can be severe: social support is critical in dealing with uncertainties and coping with negative incidents because it acts as a buffer against impactful events (Collins 2008; Waddington 1999). How organizations and the actors within them react to negative incidents also tells frontline workers how they should interpret and handle negative incidents (cf. Fleming 2020; Regehr, Goldberg, and Hughes 2002; Zacka 2017).

How the organization, manager, or colleagues deal with a citizen-related incident may matter more than the negative incident itself (Bishop, Korczynski, and Cohen 2005; Friis et al. 2020): without support, the frontline worker can feel subjected to secondary victimization. In criminology, this refers to ‘the situation in which victims feel so poorly treated by the criminal justice system that the experience was akin to being victimized all over again’ (Newburn 2017, 384). In frontline work, secondary victimization implies a breach of the psychological contract frontline workers form with others at work. Such breaches can evoke negative emotions, undermine self-esteem, cause avoidance behaviour (Davidovitz and Cohen 2021) or even moral injury (Griffin et al. 2019).

## Lived experiences of negative incidents

Table 1 shows the frequencies and impact scores of the incident (sub)categories. While the majority of incidents were filed under a single incident (sub)category (*N* = 933), many fell in two (*N* = 306), or more (three: *N* = 58; four: *N* = 5) categories. This was because some respondents mentioned several distinct incidents, meriting different labels for each. Moreover, a theoretical categorization involves ideal types, whereas real-life incidents often do not fit neatly in one category but contain elements from two or more – and interactions can evolve from one stage into another. Incident categories or subcategories that overlapped often, merit specific attention in the analysis.

**Table 1.** Frequencies and impact scores of incident categories.

				Impact scores			
Categories	Subcategories	N	%	Mean	SD	Min	Max
**A. Citizen aggression**							
A1. Physical assault and threat of assault	1. Assault	45	3.5%	72.02	22.28	7.00	100.00
	2. Threats	245	18.8%	61.50	26.38	0.00	100.00
	3. Insinuations	71	5.5%	59.13	25.32	0.00	100.00
A2. Psychological (directed) aggression	4. Yelling, cursing, spiteful comments/insults	174	13.4%	57.57	29.10	0.00	100.00
	5. Controlling/dominating behaviour/acts	123	9.4%	60.75	25.89	0.00	100.00
**B. Tragedy**	6. Facing tragedy and inability to make a difference	43	3.3%	73.26	19.65	18.00	100.00
**C. Violation of professional norms**							
C1. Instrumental violations	7. Instrumental rule-breaking	215	16.5%	56.15	26.34	0.00	100.00
	8. Accusations	120	9.2%	58.39	25.99	0.00	100.00
	9. Complaints	109	8.4%	58.84	28.31	0.00	100.00
C2. Expressive violations	10. Outbursts, unexpected show of emotions	90	6.9%	54.73	26.00	2.00	100.00
	11. ‘Assholery’	202	15.5%	53.88	27.58	0.00	100.00
**D. Absence of support**	12. “” of colleagues, manager, organization, or all of the above	59	4.5%	77.78	18.01	30.00	100.00
**Miscellaneous: Other/none**	13. Other	36	2.8%	62.47	27.68	0.00	100.00
	14. Never	207	15.9%				
**Total**		1739^2^					

The mean length of incident descriptions was 31.8 words (*SD* = 34.8). This includes incidents that were only described with a single word (e.g. ‘threat’), while the longest answer was 319 words. Most descriptions consisted of two or three sentences.

### Category A: citizen aggression

*Physical aggression* contained three subcategories: assault, threats, and insinuations. In most cases, the aggressor was the taxpayer, but there were also instances of the taxpayer’s family members, employees, or tax advisor committing acts of aggression. Assault, at 3.5% of our group a relatively small number of cases, was a fairly straightforward category. This entailed actual physical harm or attempted harm directed at the frontline worker or their property, family or colleague. It was, unsurprisingly, one of the subcategories with the highest average perceived impact (72 on the 0–100 scale). Several respondents described being traumatized and feeling unsafe and insecure long after the incident.

Common instances of physical assault entailed brief explosions of violence such as a single blow to the head, being grabbed, or a taxpayer throwing a chair or table at the tax official. Most of these incidents were obviously criminal and very serious in nature, while some were relatively mild. Examples of the latter included respondents saying that a citizen had thrown a cell phone, cup or key set at them. Examples of the former included a respondent being drugged, cases of sexual assault, taxpayers unleashing aggressive guard dogs on the tax official, or firing a bullet through the tax official’s window. Such incidents signalled that the respondent’s personal sphere was threatened:

There were two experiences in the same period (meaning: an audit at one company): First, a nail board [was placed] in front of the car so you couldn’t drive away or flee. Second, a block of concrete [was thrown] through the front window of my private address. […] I was not particularly affected, but my family was!

Threats, a wide array of incidents where physical harm was suggested, were described by 18.8% of our respondents, making it an oft-mentioned subcategory. Like with assault, some threats were relatively mild (such as the case where a taxpayer had placed a sign saying ‘death to the tax service’ in their yard, which the respondent considered equally amusing and threatening), while others were very serious (being threatened with a knife, axe or firearm). Sometimes, the threat was veiled, but so thinly that there could be no misunderstanding about the citizen’s intent:

[I received] threats by telephone by an [entrepreneur’s tax] advisor. He [said he] would come and find me. He would make sure I would be facing the highest judge on my knees. It was obvious who was meant by this highest judge.

This subcategory also included some situations without explicit threat, but where the tax official felt acute concern for their safety. These situations included cases of stalking and dealing with known or obvious criminals – who were considered unpredictable and dangerous.

A third subcategory of physical aggression was labelled ‘insinuations’, meant to cover a wide array of more indirect threats (5.5% of cases). Sometimes citizens showed malign intent through statements like ‘I would not brake if I found you in front of my car’. More often, however, citizens demonstrably knew more than they should about the private situation of the tax official or tried to find such information. While no concrete danger was present, this made the tax official feel unsafe. Other cases in this subcategory included interactions with citizens that seemed on the verge of escalating into something more serious: a large dog was present during the interaction, or an object that could serve as a weapon was in clear sight.

The second variant of citizen aggression was *psychological aggression*, which had two subcategories: first, a broad array of insults, name-calling, spiteful comments and yelling. Second, controlling or dominating behaviour. The first subcategory contained mostly expressive psychological aggression, while the second appeared instrumental in nature.

The first subcategory recurred in 13.4% of respondents’ answers. Many tax officials mentioned only in general terms being verbally abused or shouted at. Others recalled colourful language: they were labelled ‘vampires’, ‘heartless’, ‘murderers’, ‘criminals’, ‘Nazis’, ‘Gestapo’, or ‘pieces of garbage’. Some citizens made crude references to a tax official’s gender or ethnicity – several women and ethnic minority tax officials mentioned being targeted for their identity. Any purpose of these insults, other than venting frustration and ruining the tax official’s day, was impossible to discern. While few respondents were explicit about the type of impact these incidents had, many described ending the interaction or leaving the setting when they faced verbal abuse. This suggests a clear strategy in their response. While the impact scores of this subcategory were fairly average at a mean of 58, they had the highest standard deviation of all (29.1), showing that some tax officials were affected a lot, while others shrugged off such insults.

The second subcategory of psychological aggression was usually more subtle, but also more instrumental. Controlling or dominating behaviour, mentioned in 9.4% of cases, could manifest itself in various ways, but was often clearly intended to aggressively coax the tax official into a more favourable outcome:

A taxpayer who had to prove his expenses (a police officer) abused his power to pressure me into accepting the expenses. I thought someone in my family had died in an accident when he entered my office in full regalia (in uniform with walkie-talkie switched on). I did not change my position, but I was certainly cowed for a bit.

Physically controlling or dominating acts were commonly described, including citizens intimidatingly looming over the tax official, locking doors, or aggressively checking their work. Respondents recalled feeling belittled and humiliated, sometimes symbolically (a tax official was forced to take off their shoes while the citizen kept them on). More direct forms of humiliation existed too, with citizens referring to their authority as a respected and well-connected member of society’s elite (far more common in the Belgian sample than in the Dutch). Citizens threatening the tax official with lawsuits or formal complaints, or, grimly, with committing self-harm, often had similarly instrumental aims. A respondent recalled that ‘*the taxpayer suddenly opened the window and said that he would jump if I did not change the tax assessment’.*

Chi-square tests revealed significant gender and country differences in citizen aggression reports. Male tax officials were more likely than female officials to report assault (*χ^2^* (1) = 6.475, *p* = .011, *φ* = -.071) and threats (*χ^2^* (1) = 6.103, *p* = .013, *φ* = -.069). Conversely, women were more likely than men to report yelling and insults (*χ^2^* (1) = 5.545, *p* = .019, *φ* = .066) and controlling or dominating behaviour (*χ^2^* (1) = 9.362, *p* = .002, *φ* = .085). Belgian officials, too, listed yelling and insults (*χ^2^* (1) = 13.704, *p* < .001, *φ* = .103) and controlling or dominating acts (*χ^2^* (1) = 18.541, *p* < .001, *φ* = .119) more frequently than Dutch officials.

### Category B: facing tragedy and the inability to make a difference

This category occurred rarely (only 3.3% of cases), yet when mentioned its impact score was almost invariably high at 73 – surpassed only by Category D: absence of support. The two main groups included in this category involved situations where a citizen had died (often of suicide) during or briefly before or after their involvement with the tax administration, and bankruptcy of a taxpayer’s business.

The presumed suicide of a taxpayer after an audit which had ended in an agreement, but that had meant a considerable amount of due back taxes for the person concerned.

Sometimes the citizen or a family member blamed the tax official for their misfortune, and the tax official felt some responsibility. Other respondents described interactions they had with citizens who were facing serious personal issues, and while the tax official had no responsibility to address them, they made a lasting impression. While not all responses included mention of how the negative incident affected the tax official, many indicated feeling guilty, commiserating with the citizen and their family, or being otherwise emotionally affected. Often, as in the example below, respondents thought the citizen was unduly punished or deserved better.

[I had a] tax audit at a taxpayer’s home, going there twice. I was taken care of very well by the taxpayer and his wife. Unfortunately, the books did not add up, so a substantial back tax bill ensued. The taxpayer had had a heart attack several months prior. Once I told them the amount they were due, the couple was overwhelmed by emotions and I feared he would have another attack on the spot. The couple’s sadness affected me, partially because I was convinced this had all happened out of ignorance and they were really sweet people.

Chi-square tests revealed no significant gender or country differences in facing tragedy reports.

### Category C: violations of professional norms

We identified three subcategories of *instrumental violations*: instrumental rule-breaking, accusations, and complaints. These were violations of explicit norms that had a discernible purpose: norms were broken for gain (or avoiding harm) or to cast doubt on the respondent’s professional identity. Two subcategories of *expressive violations* – i.e. outbursts and assholery – however, had no tangible aim and were expressive, often irrational violations of more implicit (social) norms.

The most common subcategory (16.5% of cases) was that of instrumental rule-breaking: citizens lied, cheated, strategically drew out procedures, broke the law and/or manipulated their way out of trouble. Many respondents indicated that they were used to some measure of cheating and lying, but some circumstances could make them negative incidents. First, some accounts of instrumental rule-breaking went way beyond the commonly known boundaries of cheating. Tax officials recalled attempted bribery, made-up illnesses, taxpayers secretly recording meetings, former colleagues abusing the law as tax advisors, and other signs that suggested professional fraud. Second, cases where the tax official had expended considerable effort and goodwill, but was then deceived. Such misjudgement elicited great frustration:

I invested a lot of time, patience and energy in a taxpayer. This led to a large amount of due back taxes. Then the company went bankrupt. This gave me the feeling of having been cheated.

Particularly galling was when the citizen got away with breaking the rules – because the tax administration and/or the courts did not take sufficient action, or because the respondent had given a lying citizen the benefit of the doubt.

Next to instrumental rule-breaking, citizens tried to get their way through accusations aimed at the tax official’s professional identity. This strategy, mentioned by 9.2% of respondents, bore great similarity to the neutralization technique of ‘condemnation of the condemners’ (Sykes and Matza 1957): taxpayers attempted to shift the focus away from their own acts to the motives and behaviour of the tax official. Taxpayers or their representatives accused tax officials of racism, of taking it out on the little guy, of causing the bankruptcy of a perfectly healthy company, of being the cause of stress, sleepless nights and ill health, or of being incapable, unprofessional, or corrupt. Such accusations were often described as nasty and affected the tax official personally.

The above notwithstanding, dissatisfaction with the tax official often did not take the form of direct accusations and challenges to their professional identity, but of formal or informal complaints, as mentioned in 8.4% of cases. As with instrumental rule-breaking, filing complaints could be intended to drag out procedures and provide material gain. Sometimes complaints were virtually indistinguishable from accusations, in that they shared the function of tactically shifting the focus from the taxpayer to the tax official:

In an introductory meeting, when I asked a number of critical questions about some fiscal constructions, I sensed that the taxpayer was startled. A couple of days later a complaint was filed, in which the taxpayer completely twisted the way the meeting had occurred.

Yet the fact that a complaint was filed meant that other actors became involved: colleagues, managers, complaints boards, or judges. This complicated the situation for the tax official, who was now reliant on others to bring the situation to a close. This complexity was visible in the type of responses, where many details were provided and the feeling of being wronged was evident. Often, there was a clear overlap with Category D: absence of support.

[There was] a very tense tax audit I did with a colleague. The taxpayer, who wanted to avoid the high tax, went to complain to our management, which agreed to substantially lower the tax and therefore acted *contra legem* [against the law]. It is clear that the taxpayer was supported by management. It’s not the reaction of the taxpayer that disappointed me, although it was aggressive, but that of my management, which did not really defend its two public servants and handed the taxpayer a large gift!

Next to these instrumental and explicit subcategories of professional norm violations, two types of expressive violations were apparent. The first, unexpected emotional outbursts (6.9% of cases), consisted of taxpayers crying or showing anger, albeit not directed at the tax official. There was often a grey area where it was unclear from our data whether the citizen was merely emotional, or that there was also an element of aggression or manipulation. When such context was absent or when it was clear that the tax official believed the citizen to be genuinely distraught, incidents were coded as outbursts. Often, the respondent indicated feeling uncomfortable when such situations occurred, although sometimes they could be resolved by taking time to let the taxpayer calm down, or by good communication. This subcategory had a comparatively low average impact score (55 on the 0–100 scale).

The second expressive violation was labelled ‘assholery’, a common subcategory at 15.5% of cases. Tax officials often reported citizens showing unpleasant or uncooperative behaviour without breaking any formal rules or regulations and without clear gain. This, too, was a category with a relatively low average impact score (54). Assholery occurred when tax officials were forced to do their work in a dirty, cramped or freezing room. Sometimes, they were denied access to water or a bathroom.

First, a taxpayer let me wait out in the cold for more than an hour before they would receive me. Then, they let the audit happen in a shack without heating. In the heart of winter.

General rudeness and lack of respect were often mentioned, as were instances of tax officials being forced to wait for a citizen who was either considerably late or a no-show. Some taxpayers were drunk. Most common, however, were mentions of taxpayers refusing to cooperate – without obviously breaking the law. This made tax officials angry, annoyed, or frustrated.

Chi-square tests revealed no significant gender or country differences in violations of professional norms reports.

### Category D: absence of support

‘*[I was] accused of attempted bribery and my employer initially believed this’*. While the question explicitly inquired into interactions with taxpayers, 4.5% of respondents mentioned as their most negative experience not being supported by colleagues, managers, or the tax service as a whole when it mattered most. Remarkably, it was the incident category with the highest average impact score (78 on the 0–100 scale) and the lowest standard deviation (18.0). This impact was further illustrated by the length of their description: twice that of the average incident description at a mean of 58 words. Respondents recalled instances when colleagues or line managers had failed to act when they should have come to the respondent’s rescue when managers refused to believe their own employee, or when the organization as a whole had declined to address the respondent’s concerns.

Inherent to this category was that it was often (by 48 out of this group of 59 respondents) mentioned as a secondary element of the negative incident, exacerbating the impact of what was already an unpleasant interaction or situation. While many tax officials disliked citizens who lied or cheated, this was to some extent part of the job. What elicited a sense of betrayal was when colleagues or managers took the side of the citizen in a complaints procedure. Sometimes this was considered naiveté; other times, the result of wilful spite or corruption. A recurring pattern was the impression that it was often the absence of support that turned a bad situation into an actual negative incident. This was the case when a citizen who refused to deal with a female tax official was accommodated and a male colleague was sent instead. The respondent experienced this as deeply unjust. Similarly, multiple respondents described being dragged into difficult legal procedures by citizens, without receiving even a modicum of support from their organization.

Chi-square tests revealed no significant gender or country differences in absence of support reports.

### Miscellaneous: Other/none

Some answers (2.8%) featured negative incidents that were not readily categorizable, including answers that were conceptually different from the preceding categories. First, there were remarks concerning general working conditions and reorganizations within the tax administration that held no reference to citizens. A second group described situations where the tax official dealt with real criminals while no threatening element existed. Finally, some tax officials mentioned situations where they had made a mistake in the course of their work with consequences for the taxpayer, like a calculation error, which they felt bad about. In addition, a substantial share of respondents (15.9% of cases) indicated they never had seriously bad interactions with citizens or that nothing specific came to mind.

## Understanding patterns in negative incidents

Several patterns emerge from the analysis concerning the nature, direction, and impact of negative incidents. In terms of incident nature, descriptions of workplace aggression and tragedy in frontline work emphasize the incidents themselves, with aspects that are *intrinsic* to the bureaucratic encounter turning it into a negative incident. Contrastingly, violations of professional norms and the absence of support rely on more complex considerations of *context* and sense-making to uncover what makes an encounter a negative incident. After all, while aggression in public sphere interactions is broadly condemned (cf. Elias 2000), what counts as a violation of a professional norm is flexible. Lying, for instance, may in some social or indeed professional settings, such as advertising, be part of the dominant norm (Kocher, Schudy, and Spantig 2018). Hence, these negative incidents do not exist in isolation but are socially constructed by virtue of setting, interpretation and context.

**Figure 1.**
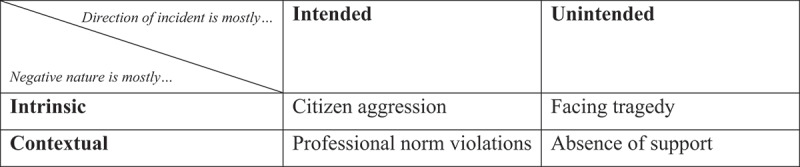
Patterns in frontline negative incident categories.

A second pattern in negative incidents is their direction. Is their occurrence the direct, *intended* result of an act or statement, or rather an *unintended* coincidence or by-product of another mechanism? Citizen aggression and most forms of professional norm violations are intended, but tragedy is not – nor are most occurrences of absence of support. Figure 1 summarizes these patterns in incident categories along two dimensions. It is noteworthy that negative incidents that contain a measure of intent may be expressive (a citizen wants to vent their frustration at the tax official) or instrumental (a citizen wants to gain something). Whether or not an intention is expressive or instrumental is usually known for professional norm violations, since these are contextual in nature. Because we understand the context, we know whether the citizen wants to gain something. However, the intention is often unknown for many forms of aggression as these incidents’ negative nature is intrinsic.

When it comes to impact patterns, finally, highly impactful negative incidents exist in each discernible category within the incident recollections. However, the emotions connected to an incident and their consequences differ across (sub)categories. Physical aggression mainly causes fear and feelings of unsafety and nervosity. Psychological aggression in many cases leads to respondents feeling belittled or humiliated and ending the interaction as a strategic response. Tragedy is often accompanied by guilt, while violations of professional norms are shown to elicit frustration and annoyance. Absence of support was often detailed in long sequences where no explicit mention of impact was given, but a sense of betrayal was clear.

## Discussion

This study developed a framework for understanding frontline negative incidents using an abductive approach that integrated theoretical notions from various literature streams with empirical insights drawn from 1302 negative incidents experienced by Dutch and Belgian frontline tax officials. This resulted in a framework with four overarching categories: A) citizen aggression; B) facing tragedy; C) violations of professional norms; D) absence of support. Each of these categories included deeply impactful negative incidents, yet the source and type of impact differed substantially.

Our framework has various theoretical implications. First, while most scholarly and policy attention focuses on aggression incidents (e.g. Friis et al. 2020; Van Reemst and Fischer 2019), *a more holistic perspective on frontline tensions* can be fostered by placing the concept of frontline negative incidents centre stage. Aggression forms merely a segment of a spectrum of incident types and should be understood as such.

Consequently, our framework reveals important *oversights* in how different academic disciplines understand frontline negative incidents. For instance, public administration research into frontline incidents has paid scant attention to workplace support (cf. Keulemans and Van de Walle 2020a; Raaphorst 2017). Intuitively, that makes sense; workplace support is not a part of those interactions. Yet our study has shown that absent support can act as a *catalyst* that intensifies the impact of negative interactions with citizens (cf. Shenk and Fruzzetti 2011), turning that interaction into an incident. This oversight aligns with broader trends in public administration, where the role of social context and social processes is often overlooked (see Raaphorst 2017).

Our framework additionally demonstrates that incident types *differ along multiple dimensions*. First, the negative meaning of the incident can be inherent to the incident, or constructed by taking its context into account. Second, negative incidents where harm was intended by a perpetrator can be distinguished from those where there is no such intent. Aggression incidents are usually intended and their negative impact is embedded in the interaction itself. However, many of the most impactful negative incidents portrayed in this study are either unintentional in direction, contextual in nature, or both. Their type of impact and the emotions they trigger may also differ. Lack of awareness of these incident patterns means that negative incidents are easily misunderstood by scholars, public agencies, and public managers.

Connecting these insights, we have seen that frontline incident research thus far has primarily focused on specific incident types and their impact; whether they be workplace aggression and fear or trauma (e.g. Keesman and Weenink 2020; Rogers and Kelloway 1997), professional norm violations and expanding professional (emotional) resources (e.g. Davidovitz and Cohen 2021), or witnessing tragedy and feelings of guilt and exhaustion (e.g. Regehr, Goldberg, and Hughes 2002). Our study suggests that much can be gained by combining these insights and research traditions to advance our understanding of phenomena of interest to various academic disciplines.

Frontline coping serves as a notable illustration. Our study highlights that physical aggression induces fear, while tragedy evokes guilt. Fear triggers more acute physiological reactions and is verbalized more often than guilt (Matsumoto et al. 1988). Different stressors evoke different coping strategies (Keay and Bandler 2001), which suggests that aggression incidents and tragedy incidents shape frontline coping in different ways. Understanding how different incident types and their specific impact relate to various coping strategies could offer insight into the boundary conditions of frontline workers’ coping behaviour.

Some study limitations require consideration. First, to collect incident data we used a retrospective research method that relies on memory processes susceptible to recollection and reconstruction bias (see Erdfelder and Buchner 1998). This raises the question of what implications memory bias may have had for the negative incident framework. However, not objective incidents but the *meaning* of those incidents was our research subject. Frontline workers construct meaning by making sense of their experiences (cf. Merriam and Cafferella 1999, 260). How they recall incidents reflects how those incidents actually took place in the real world *for them*. Those recollections have real-world consequences, irrespective of any bias (Thomas and Thomas 1928, 527), that are critical to understanding frontline negative incidents.

Second, we focused empirically on tax officials. Although citizens have been found to behave fairly similarly towards regulatory and service bureaucrats (Nielsen, Nielsen, and Bisgaard 2021), there will be differences with other frontline professions. For instance, with regard to how often they will encounter human tragedy or whether they interact with clients on-site or in the office. Similarly, *within* incident categories the incident types may vary between professions; police officers may identify an ‘asshole’ by their big mouth (Van Maanen 1978), while tax officials recognize one when they are forced to wait in a room without heating.

Expanding on this, Guy, Mastracci, and Yang (2019) describe how contextual, cultural, and social differences between countries shape public servants’ expectations, and consequently, interpretations of citizen behaviour. Within incident categories, there were some differences. Belgian respondents more often reported a form of psychological aggression as their most negative incident than Dutch respondents. We may wonder whether those incidents are more common in Belgium than in the Netherlands, or whether such incidents are more likely to be interpreted as particularly negative in Belgium. Considering the similar tasks and work environments of tax officials in the two countries, social norms are more likely than structural differences to account for the discrepancy; national cultural characteristics, such as higher emphasis on power distance and less focus on consensus in Belgium, could make the occurrence of psychological aggression more likely, while comparatively lower individualism and higher uncertainty avoidance in the country could contribute to behaviour being more likely to be understood as psychologically aggressive in nature (cf. Hofstede 2001). Despite these differences, the conceptual boundaries between incident categories were fairly clear in this study, and while our framework holds for frontline tax officials in two countries, we cannot ignore the possibility that these may be blurred or have to be drawn differently in different contexts.

This study set out to provide building blocks for a theory of frontline negative incidents. Reasoning from the study findings, we can infer that such a theory will consist of five building blocks. First, we must recognize that frontline workers in the course of their work can have various interactions that are experienced as very negative and that these interactions can profoundly affect the worker in different ways. Second, the negative nature of these incidents can be intended or unintended, and intrinsic or contextual. Third, physical violence, absence of support, and witnessing tragedy generally have the highest impact on the frontline worker, but do so through different mechanisms. Fourth, the frequency, nature and impact of negative incidents likely vary across professions and contexts. Fifth, the occurrence of negative incidents helps explain problems with frontline work (such as stress, cynicism or burnout), but these problems also may help explain the future occurrence of negative incidents.

These building blocks primarily address the concept of frontline negative incidents and their underlying principles. A second step in theory development would entail gaining insight into how this concept relates to other phenomena (see Emerson 2022). This includes the antecedents and consequences of frontline negative incidents and their impact.

As an initial attempt at this, our analysis revealed that male officials were more likely than female officials to report assault or threats as their most negative incident, while female officials were more likely to list instances of psychological aggression. This suggests gender differences in the occurrence and interpretation of negative incidents that may be explained by societal gender norms being perpetuated in the bureaucratic encounter. Physical aggression towards men is deemed more socially acceptable than aggression against women (Graham and Wells 2001, 596), which turns male frontline workers into more likely victims of those incidents. Simultaneously, these norms may drive citizens who intend to inflict harm on a female frontline worker to resort to psychological aggression. The latter may be legitimized by gender norms that attribute less status, power, and assertiveness to women than to men (e.g. Eagly and Chrvala 1986; Elliott and Smith 2004). Female frontline workers’ authority may then be experienced as a defiance of female gender stereotypes, making them vulnerable to backlash in various forms (cf. Chakraborty and Serra 2023). More research into how such mechanisms relate to frontline negative incidents can further contribute to a theory of frontline negative incidents.

## Conclusion

Directing attention to frontline workers’ lived experiences, this study developed a framework for understanding negative incidents that sheds light on their diversity and impact. It demonstrates that empirical studies on frontline workers’ actual rather than hypothetical interactions with citizens are critical to understanding what happens in bureaucratic encounters – and how these encounters are made sense of.

Our framework has multiple practical implications. Its more holistic perspective on frontline negative incidents invites public agencies to prioritize non-aggression incidents. For instance, workplace support is often overlooked, while research indicates a lack of social support across all organizational levels following negative incidents (Davidovitz and Cohen 2021; Tzafrir, Enosh, and Gur 2015). Organizations that neglect to properly address incidents contribute to incident invisibility, fostering underreporting, denial or victim-blaming (Bishop, Korczynski, and Cohen 2005; Skolnick 1966). Recognizing other incident types may also help resolve discrepancies between the incidents public managers assume merit organizational support and those actually experienced as most impactful (cf. Bishop, Korczynski, and Cohen 2005; Regehr, Goldberg, and Hughes 2002), thus better enabling managers to safeguard frontline workers’ well-being.

A dimensional structure to negative incidents suggests that policies aimed at prevention and dealing with their aftermath should develop differentiated approaches to separate incident types. Citizens who use (psychological) aggression instrumentally, causing the frontline worker to feel afraid or humiliated, will, for instance, require very different measures and policies to counter than the emotional outburst of a desperate business owner that causes the frontline worker to lose control over the interaction. The incident may play out in comparable ways and have a similar impact. However, in the former case, public agencies should probably focus on strict punishment of aggressive citizens and a rule-based approach, whereas the latter situation may rather stimulate agencies to rethink the way frontline workers could handle difficult interaction situations in order to mitigate and minimize harm.

Multiple avenues for future research arise from our study. First, future survey research quantifying the occurrence of incident categories is welcomed. Quantification may stimulate public agencies and public managers to prioritize and take more defined steps towards dealing with incidents and could provide insight into at-risk groups or professions. Second, our survey methodology precluded follow-up questions on incident recollections. Gaining further insight into how frontline workers make sense of incident impact would be particularly valuable. In-depth interviews, diary studies, or observational research could offer valuable approaches. Finally, we encourage exploring the generalizability of our framework to other frontline professions and cultural settings. Such explorations could foster further development and discussion on the nature and consequences of frontline negative incidents, to the benefit of scholarship and practice.

## Data Availability

The data that support the findings of this study are not publicly available due to privacy and ethical restrictions.

## Notes

1. One of the five Dutch tax regions participated in a prior pilot study and was therefore excluded from our survey.

2. The total number of incidents exceeds 1302 because incidents could be categorized into multiple categories.

## Appendix I: Full negative incident framework (v3)

**Table:**

**A.****Citizen aggression** **A1. *Physical assault and threat of assault*** **1. Assault** (actual or attempted physical harm; any force directed at respondent or respondent’s property, colleague or family). **2. Threats** (direct or very thinly veiled threats; situations where respondent is clearly afraid for their physical safety; weapons explicitly shown; aggressive stalking). **3. Insinuations** (indirect threats; weapons or dogs present but not emphasized; veiled or implied threats). **A2. *Psychological (directed) aggression*** **4. Yelling, cursing, spiteful comments/insults** (intentional, aimed at respondent; includes racist or sexist remarks – so, psychological aggression without obvious instrumental purpose). **5. Controlling/dominating behaviour/acts** (next to physically imposing acts, such as locking doors or aggressively checking respondent’s work, also includes: discriminatory behaviour; reference to authority; condescension; threatening self-harm, lawsuits or complaints – so, psychological aggression with instrumental purpose). **B.** **Tragedy** **6. Facing tragedy and inability to make a difference** **C. Violation of professional norms** **C1. *Instrumental violations*** **7. Instrumental rule-breaking – lies/dishonesty, not meeting obligations, manipulation, bribery** (includes fraud or corruption; also includes dragging out procedures when explicitly of instrumental use [‘dragging their feet’] and instrumental use of emotion [‘crying for show’]). **8. Accusations** (in respondent’s face; aimed at professional identity of respondent). **9. Complaints** (either formal or informal, organization/supervisor/formal system). **C2. *Expressive violations*** **10. Outbursts, unexpected show of emotions** (undirected; includes ‘anger’ if no context is given as to its direction; crying if not clearly for manipulative purposes). **11. ‘Assholery’ – rudeness, avoidance, refusal to cooperate, being unwelcome** (citizen is ungovernable, but primarily because they are assholes, not because they have discernible goals (in the latter case: 5 or 7) – overlap can exist). **D. Absence of support** **12. Absence of support of colleagues, supervisor, organization, or all the above** (when related to contact with citizens; not when only general remarks about things like lack of career opportunities). **Miscellaneous: Other/none** **13. Other** **14. Never**

## References

[cit0001] Bishop, Vicky, Marek Korczynski, and Laurie Cohen. 2005. “The Invisibility of Violence: Constructing Violence Out of the Job Centre Workplace in the UK.” *Work, Employment and Society* 19 (3): 583–602. 10.1177/0950017005055671.

[cit0002] Bittner, Egon. 1967. “Policing on Skid-Row: A Study of Peace Keeping.” *American Sociological Review* 32 (5): 699–715. 10.2307/2092019.

[cit0003] Björk, Micael. 2008. “Fighting Cynicism. Some Reflections on Self-Motivation in Police Work.” *Police Quarterly* 11 (1): 88–101. 10.1177/1098611107309010.

[cit0004] Blau, Peter M. 1960. “Orientation Towards Clients in a Public Welfare Agency.” *Administrative Science Quarterly* 5 (3): 341–361. 10.2307/2390661.

[cit0005] Bowen, Glenn A. 2006. “Grounded Theory and Sensitizing Concepts.” *International Journal of Qualitative Methods* 5 (3): 12–23. 10.1177/160940690600500304.

[cit0006] Braithwaite, Valerie. 2009. *Defiance in Taxation and Governance Resisting and Dismissing Authority in a Democracy*. Northampton, MA: Edward Elgar Publishing, Inc.

[cit0007] Chakraborty, Priyanka, and Danila Serra. 2023. “Gender and Leadership in Organisations: The Threat of Backlash.” *The Economic Journal* 134 (660): 1401–1430. 10.1093/ej/uead110.

[cit0008] Coetzee, Siedine Knobloch, and Hester C. Klopper. 2010. “Compassion Fatigue within Nursing Practice: A Concept Analysis.” *Nursing & Health Sciences* 12 (2): 235–243. 10.1111/j.1442-2018.2010.00526.x. 20602697

[cit0009] Collins, Stewart. 2008. “Statutory Social Workers: Stress, Job Satisfaction, Coping, Social Support and Individual Differences.” *British Journal of Social Work* 38 (6): 1173–1193. 10.1093/bjsw/bcm047.

[cit0010] Crank, John P. 2014. *Understanding Police Culture*. New York: Routledge.

[cit0011] Davidovitz, Maayan, and Nissim Cohen. 2021. “‘I Have Learned My Lesson’: How Clients’ Trust Betrayals Shape the Future Ways in which Street‐Level Bureaucrats Cope with Their Clients.” *Public Administration* 101 (1): 335–351. 10.1111/padm.12769.

[cit0012] De Boer, Noortje. 2021. “The (Un)intended Effects of Street-Level Bureaucrats’ Enforcement Style: Do Citizens Shame or Obey Bureaucrats?” *Public Policy and Administration* 36 (4): 452–475. 10.1177/0952076720905005.

[cit0013] Dubois, Vincent. 2010. *The Bureaucrat and the Poor: Encounters in French Welfare Offices*. Farnham, UK: Ashgate Publishing Limited.

[cit0014] Eagly, Alice H., and Carole Chrvala. 1986. “Sex Differences in Conformity: Status and Gender Role Interpretations.” *Psychology of Women Quarterly* 10 (3): 203–220. 10.1111/j.1471-6402.1986.tb00747.x.

[cit0015] Edvardsson, Bo, and Inger Roos. 2001. “Critical Incident Techniques: Towards a Framework for Analyzing the Criticality of Critical Incidents.” *International Journal of Service Industry Management* 12 (3): 251–268. 10.1108/EUM0000000005520.

[cit0016] Elias, Norbert. 2000. *The Civilizing Process. Sociogenetic and Psychogenetic Investigations*. Rev. ed. Malden, MA: Blackwell.

[cit0017] Elliott, James R., and Ryan A. Smith. 2004. “Race, Gender, and Workplace Power.” *American Sociological Review* 69 (3): 365–386. 10.1177/000312240406900303.

[cit0018] Emerson, Kirk. 2022. “On Theory and Theory Building in Public Administration.” *Perspectives on Public Management and Governance* 5 (1): 3–10. 10.1093/ppmgov/gvab032.

[cit0019] Erdfelder, Edgar, and Axel Buchner. 1998. “Decomposing the Hindsight Bias: A Multinomial Processing Tree Model for Separating Recollection and Reconstruction in Hindsight.” *Journal of Experimental Psychology: Learning, Memory & Cognition* 24 (2): 387–414. 10.1037/0278-7393.24.2.387.

[cit0020] Figley, Charles R. 1995. “Compassion Fatigue as Secondary Traumatic Stress Disorder: An Overview.” In *Compassion Fatigue. Coping with Secondary Traumatic Stress Disorder in Those Who Treat the Traumatized*, edited by Charles R. Figley, 1–20. New York, NY: Routledge.

[cit0021] Flanagan, John C. 1954. “The Critical Incident Technique.” *Psychological Bulletin* 51 (4): 327–358. 10.1037/h0061470. 13177800

[cit0022] Fleming, Casey J. 2020. “Prosocial Rule Breaking at the Street Level: The Roles of Leaders, Peers, and Bureaucracy.” *Public Management Review* 22 (8): 1191–1216. 10.1080/14719037.2019.1619817.

[cit0023] Friis, Camilla B., Lasse S. Liebst, Richard Philpot, and Marie R. Lindegaard. 2020. “Ticket Inspectors in Action: Body-Worn Camera Analysis of Aggressive and Nonaggressive Passenger Encounters.” *Psychology of Violence* 10 (5): 483–492. 10.1037/vio0000276.

[cit0024] Graham, Kathryn, and Samantha Wells. 2001. “The Two Worlds of Aggression for Men and Women.” *Sex Roles* 45 (9–10): 595–622. 10.1023/A:1014811624944.

[cit0025] Griffin, Brendon J., Natalie Purcell, Kristine Burkman, Brett T. Litz, Craig J. Bryan, Martha Schmitz, Claudia Villierme, Jessica Walsh, and Shira Maguen. 2019. “Moral Injury: An Integrative Review.” *Journal of Traumatic Stress* 32 (3): 350–362. 10.1002/jts.22362. 30688367

[cit0026] Grove, Richard W. 1988. “An Analysis of the Constant Comparative Method.” *International Journal of Qualitative Studies in Education* 1 (3): 273–279. 10.1080/0951839900030105a.

[cit0027] Guy, Mary E., Sharon H. Mastracci, and Seung-Bum Yang, eds. 2019. *The Palgrave Handbook of Global Perspectives on Emotional Labor in Public Service*. Cham, Switzerland: Palgrave Macmillan.

[cit0028] Handy, Jocelyn, and Kirsty Ross. 2005. “Using Written Accounts in Qualitative Research.” *South Pacific Journal of Psychology* 16 (1): 40–47. 10.1017/S0257543400000067.

[cit0029] Hofstede, Geert H. 2001. *Comparing Values, Behaviors, Institutions and Organizations Across Nations*. 2nd ed. Thousand Oaks, CA: Sage.

[cit0030] Jackson, Kristin M., and William MK Trochim. 2002. “Concept Mapping as an Alternative Approach for the Analysis of Open-Ended Survey Responses.” *Organizational Research Methods* 5 (4): 307–336. 10.1177/109442802237114.

[cit0031] Keay, Kevin A., and Richard Bandler. 2001. “Parallel Circuits Mediating Distinct Emotional Coping Reactions to Different Types of Stress.” *Neuroscience & Biobehavioral Reviews* 25 (7–8): 669–678. 10.1016/S0149-7634(01)00049-5. 11801292

[cit0032] Keesman, Laura D., and Don Weenink. 2020. “Bodies and Emotions in Tense and Threatening Situations.” *Journal of Social Work* 20 (2): 173–192. 10.1177/1468017318795726.

[cit0033] Kelle, Udo. 1995. “Theories as Heuristic Tools in Qualitative Research.” In *Openness in Research: The Tension Between Self and Other*, edited by Ilja Maso, Paul Atkinson, Sara Delamont, and Jef Verhoeven, 33–50. Assen, The Netherlands: Van Gorcum.

[cit0034] Keulemans, Shelena. 2020. “Understanding Street-Level Bureaucrats’ Attitude Towards Clients: A Social Psychological Approach.” PhD diss., Erasmus University Rotterdam.

[cit0035] Keulemans, Shelena, and Steven Van de Walle. 2020a. “Street-Level Bureaucrats’ Attitude Toward Clients: A Study of Work Group Influence in the Dutch and Belgian Tax Administration.” *Public Performance & Management Review* 43 (2): 334–362. 10.1080/15309576.2019.1697303.

[cit0036] Keulemans, Shelena, and Steven Van de Walle. 2020b. “Understanding Street-Level Bureaucrats’ Attitude Towards Clients: Towards a Measurement Instrument.” *Public Policy and Administration* 35 (1): 84–113. 10.1177/0952076718789749.

[cit0037] Kocher, Martin G., Simeon Schudy, and Lisa Spantig. 2018. “I Lie? We Lie! Why? Experimental Evidence on a Dishonesty Shift in Groups.” *Management Science* 64 (9): 3995–4008. 10.1287/mnsc.2017.2800.

[cit0038] Krosnick, Jon A., Charles M. Judd, and Bernd Wittenbrink. 2005. “The Measurement of Attitudes.” In *The Handbook of Attitudes*, edited by Dolores Albarracín, T. Jonhson Blair, and P. Zanna Mark, 21–78. Mahwah, NJ: Lawrence Erlbaum Associates, Publishers.

[cit0039] Lipsky, Michael. 2010. *Street-Level Bureaucracy: Dilemmas of the Individual in Public Service*. 30th anniversary expanded ed. New York, NY: Russell Sage Foundation.

[cit0040] Matsumoto, David, Tsutomu Kudoh, Klaus Scherer, and Harald Wallbott. 1988. “Antecedents of and Reactions to Emotions in the United States and Japan.” *Journal of Cross-Cultural Psychology* 19 (3): 267–286. 10.1177/0022022188193001.

[cit0041] Maynard-Moody, Steven W., and Michael C. Musheno. 2003. *Cops, Teachers, Counselors: Stories from the Front Lines of Public Service*. Ann Arbor, MI: University of Michigan Press.

[cit0042] Merriam, Sharan B., and Rosemary S. Cafferella. 1999. “Key Theories of Learning.” In *Learning in Adulthood: A Comprehensive Guide*, edited by Sharan B. Merriam and Rosemary S. Cafferella, 248–266. 2nd ed. San Francisco, CA: Jossey-Bass Publishers.

[cit0043] Montgomery, Andrew C., and Kathleen S. Crittenden. 1977. “Improving Coding Reliability for Open-Ended Questions.” *Public Opinion Quarterly* 41 (2): 235–243. 10.1086/268378.

[cit0044] Mousa, Mohamed, Shlomo Tarba, Ahmad Arslan, and Sir Cary Cooper. 2023. “When Extreme Work Becomes the Norm: An Exploration of Coping Strategies of Public Sector Nurses.” *Public Management Review*. Advance online publication. 1–20. 10.1080/14719037.2023.2246493.

[cit0045] Muir, William K. 1977. *Police: Streetcorner Politicians*. Chicago, IL: University of Chicago Press.

[cit0046] Newburn, Tim. 2017. *Criminology*. 3rd ed. Abingdon: Routledge.

[cit0047] Nielsen, Helle Ørsted, and Vibeke Lehmann Nielsen. 2022. “Different Encounter Behaviors: Businesses in Encounters with Regulatory Agencies.” *Regulation & Governance* 17 (1): 61–82. 10.1111/rego.12455.

[cit0048] Nielsen, Vibeke Lehmann, Helle Ørsted Nielsen, and Mette Bisgaard. 2021. “Citizen Reactions to Bureaucratic Encounters: Different Ways of Coping with Public Authorities.” *Journal of Public Administration Research & Theory* 31 (2): 381–398. 10.1093/jopart/muaa046.

[cit0049] Paoline, Eugene. A., III. 2003. “Taking Stock: Towards a Richer Understanding of Police Culture.” *Journal of Criminal Justice* 31 (3): 199–214. 10.1016/S0047-2352(03)00002-3.

[cit0050] Papouli, Eleni. 2016. “Using the Critical Incident Technique (CIT) to Explore How Students Develop Their Understanding of Social Work Values and Ethics in the Workplace During Their Final Placement.” *Journal of Social Work Values and Ethics* 13 (2): 56–72.

[cit0051] Popping, Roel. 2015. “Analyzing Open-Ended Questions by Means of Text Analysis Procedures.” *Bulletin of Sociological Methodology/Bulletin de Méthodologie Sociologique* 128 (1): 23–39. 10.1177/0759106315597389.

[cit0052] Raaphorst, Nadine J. 2017. “Uncertainty in Bureaucracy: Toward a Sociological Understanding of Frontline Decision Making.” PhD diss., Erasmus University Rotterdam.

[cit0053] Raaphorst, Nadine J. 2018. “How to Prove, How to Interpret and What to Do? Uncertainty Experiences of Street-Level Tax Officials.” *Public Management Review* 20 (4): 485–502. 10.1080/14719037.2017.1299199.

[cit0054] Regehr, Cheryl, Gerald Goldberg, and Judy Hughes. 2002. “Exposure to Human Tragedy, Empathy, and Trauma in Ambulance Paramedics.” *The American Journal of Orthopsychiatry* 72 (4): 505–513. 10.1037//0002-9432.72.4.505. 15792036

[cit0055] Richardson, Rudy, and Eric Hans Kramer. 2006. “Abduction as the Type of Inference That Characterizes the Development of a Grounded Theory.” *Qualitative Research* 6 (4): 497–513. 10.1177/1468794106068019.

[cit0056] Rogers, Kimberley-Ann, and E. Kevin Kelloway. 1997. “Violence at Work: Personal and Organizational Outcomes.” *Journal of Occupational Health Psychology* 2 (1): 63–71. 10.1037//1076-8998.2.1.63. 9552280

[cit0057] Sausdal, David. 2018. “Pleasures of Policing: An Additional Analysis of Xenophobia.” *Theoretical Criminology* 22 (2): 226–242. 10.1177/1362480617707947.

[cit0058] Savaya, Riki, Fiona Gardner, and Dorit Stange. 2011. “Stressful Encounters with Social Work Clients: A Descriptive Account Based on Critical Incidents.” *Social Work* 56 (1): 63–71. 10.1093/sw/56.1.63. 21314072

[cit0059] Schat, Aaron C. H., and Michael R. Frone. 2011. “Exposure to Psychological Aggression at Work and Job Performance: The Mediating Role of Job Attitudes and Personal Health.” *Work & Stress* 25 (1): 23–40. 10.1080/02678373.2011.563133. 21643471 PMC3105890

[cit0060] Schat, Aaron C. H., and E. K. Kelloway. 2005. “Workplace Aggression.” In *Handbook of Work Stress*, edited by J. Barling, E. K. Kelloway, and M. R. Frone, 189–218. Thousand Oaks, CA: Sage Publications.

[cit0061] Schat, Aaron C. H., and E. Kevin Kelloway. 2003. “Reducing the Adverse Consequences of Workplace Aggression and Violence: The Buffering Effects of Organizational Support.” *Journal of Occupational Health Psychology* 8 (2): 110–122. 10.1037/1076-8998.8.2.110. 12703877

[cit0062] Schwarz, Norbert. 2008. “Attitude Measurement.” In *Attitudes and Attitude Change*, edited by William D. Crano and Radmila Prislin, 41–60. London, UK/New York, NY: Psychology Press. 10.4324/9780203838068.

[cit0063] Sciepura, Brenda, and Elizabeth Linos. 2022. “When Perceptions of Public Service Harms the Public Servant: Predictors of Burnout and Compassion Fatigue in Government.” *Review of Public Personnel Administration* 44 (1): 116–138. 10.1177/0734371X221081508.

[cit0064] Shenk, Chad E., and Alan E. Fruzzetti. 2011. “The Impact of Validating and Invalidating Responses on Emotional Reactivity.” *Journal of Social & Clinical Psychology* 30 (2): 163–183. 10.1521/jscp.2011.30.2.163.

[cit0065] Sivis-Cetinkaya, Rahsan. 2015. “Ethical Dilemmas of Turkish Counsellors: A Critical Incidents Study.” *British Journal of Guidance & Counselling* 43 (4): 476–491. 10.1080/03069885.2014.987726.

[cit0066] Skolnick, Jerome H. 1966. *Justice without Trial. Law Enforcement in Democratic Society*. New York: Wiley.

[cit0067] Sykes, Gresham M., and David Matza. 1957. “Techniques of Neutralization: A Theory of Delinquency.” *American Sociological Review* 22 (6): 664–670. 10.2307/2089195.

[cit0068] Thomas, William I., and Dorothy S. Thomas. 1928. *The Child in America: Behavior Problems and Programs*. New York, NY: A. A. Knopf.

[cit0069] Thompson Burdine, Julie, Sally Thorne, and Gurjit Sandhu. 2021. “Interpretive Description: A Flexible Qualitative Methodology for Medical Education Research.” *Medical Education* 55 (3): 336–343. 10.1111/medu.14380. 32967042

[cit0070] Thorne, Sally, Sheryl Reimer-Kirkham, and Katherine O’Flynn-Magee. 2004. “The Analytic Challenge in Interpretive Description.” *International Journal of Qualitative Methods* 3 (1): 1–11. 10.1177/160940690400300101.

[cit0071] Tzafrir, Shay S., Guy Enosh, and Amit Gur. 2015. “Client Aggression and the Disenchantment Process Among Israeli Social Workers: Realizing the Gap.” *Qualitative Social Work* 14 (1): 64–85. 10.1177/1473325013509827.

[cit0072] Van der Ploeg, Eleonore, Sasja M. Dorresteijn, and Rolf J. Kleber. 2003. “Critical Incidents and Chronic Stressors at Work: Their Impact on Forensic Doctors.” *Journal of Occupational Health Psychology* 8 (2): 157–166. 10.1037/1076-8998.8.2.157. 12703881

[cit0073] Van de Walle, Steven, and Nadine J. Raaphorst, eds. 2019. *Inspectors and Enforcement at the Front Line of Government*. Cham, Switzerland: Palgrave Macmillan.

[cit0074] Van Maanen, John. 1978. “The Asshole.” In *Policing: A View from the Street*, edited by Peter K. Manning and John van Maanen, 221–238. Santa Monica, CA: Goodyear Publishing.

[cit0075] Van Reemst, Lisa, and Tamar F. Fischer. 2019. “Experiencing External Workplace Violence: Differences in Indicators Between Three Types of Emergency Responders.” *Journal of Interpersonal Violence* 34 (9): 1864–1889. 10.1177/0886260516657913. 27413089

[cit0076] Waddington, Peter A. J. 1999. “Police(canteen) Sub-Culture. An Appreciation.” *The British Journal of Criminology* 39 (2): 287–309. 10.1093/bjc/39.2.287.

[cit0077] Zacka, Bernardo. 2017. *When the State Meets the Street: Public Service and Moral Agency*. Cambridge, MA: Harvard University Press.

